# A randomized controlled trial for evaluating an occupational therapy self management intervention in adults with type 2 diabetes

**DOI:** 10.1038/s41598-023-37231-9

**Published:** 2023-06-22

**Authors:** Maryam Binesh, Narges Shafaroodi, Majid Mirmohammadkhani, Rokhsareh Aghili, Fatemeh Motaharinezhad, Mahnoosh Khanipour, Afsoon Hassani Mehraban

**Affiliations:** 1 Department of Occupational Therapy, Neuromuscular Rehabilitation Research Center, Semnan University of Medical Sciences, Semnan, Iran; 2 Department of Occupational Therapy, Rehabilitation Research Center, School of Rehabilitation Sciences, Iran University of Medical Sciences, Tehran, Iran; 3 Social Determinants of Health Research Center, Semnan University of Medical Sciences, Semnan, Iran; 4 Endocrinology; Endocrine Research Center, Institute of Endocrinology and Metabolism, Iran University of Medical Sciences, Tehran, Iran; 5 Department of Occupational Therapy, School of Paramedical Sciences, Zanjan University of Medical Sciences, Zanjan, Iran; 6 Rehabilitation Research Center, Occupational Therapy Department, School of Rehabilitation Sciences, Iran University of Medical Sciences, Tehran, Iran

**Keywords:** Endocrinology, Health care, Medical research

## Abstract

This study evaluated the efficacy of the Occupational Therapy Diabetes Self-Management intervention (OTDSM) to enhance glycemic stability and self-management skills in people with diabetes type-2. Based on this single-blind randomized trial, 30 subjects with diabetes type-2 were assigned to two groups of intervention and control. The intervention group received a 10-week program, consisting of four group visits and six individualized sessions. The control group received an individual session and three weekly phone calls. The primary study outcome, blood hemoglobin A1C, was measured before and three months after the study. The secondary outcome was assessed in terms of the participants’ self-management behaviors, self-efficacy, diabetes distress, depressive symptoms, and performance and satisfaction with daily activities. These outcomes were evaluated three times: before, one month into, and three months after the study. The study findings demonstrated significant differences between the two groups in the hemoglobin A1C levels, self-management behaviors, self-efficacy, and performance and satisfaction with daily routines after the intervention (P < 0.05). No significant differences existed between the groups for the extent of diabetes distress and depressive symptoms. Inclusion of occupational therapy protocol into the plan of care for people with diabetes can improve health outcomes by promoting their routine participation in self-management activities.

## Introduction

Diabetes Type-2, a disease caused by the body's failure to secrete or utilize insulin^1^, is a major health concern for over 400 million adults worldwide^2^. The growing prevalence of this disease is associated with several debilitating complications that affect people's lives in many ways and impose a huge healthcare burden on governments. Therefore, diabetes management is of prime concern for the healthcare systems globally^1^.

Self-management is part of an effective solution to the management of many chronic diseases, including diabetes. Self-management is a process, requires us to advocate and promote a healthy lifestyle. They should actively participate in efforts related to the management of the diabetes symptoms, complications, treatments, and physical and psychosocial consequences of the disease^3^. To empower people with diabetes in this process, health professionals must develop and implement self-management ideas in an interdisciplinary approach. By expanding intrapersonal capacities and involving the social environment and interpersonal relationships, many programs have achieved desirable outcomes^4^. However, available interventions almost missed to encompass and integrate with healthcare activities with people getting used to daily routines in their real-life context to establish a healthy lifestyle for themselves and others^4^.

Occupational therapists are health professionals with expertise in empowering people to participate in meaningful daily activities. Using a holistic approach, occupational therapists can consider various patients and contextual factors, and the patterns of one's daily activities to draw unique treatment approaches and guide people toward regular participation in self-management^5^.

Currently, there is some evidence available on diabetes self-management (DSM) and related interventions based on occupational therapy perspectives. These studies explored ways to improve blood glucose levels^6–8^, self-management behaviors, and self-efficacy^9,10^. Future research is warranted to enrich the existing approaches on the effective role of occupational therapy and develop innovative DSM protocols.

Occupational therapy diabetes self-management intervention (OTDSM) is a program developed based on available occupational therapy evidence and practice framework. The intervention, aimed to facilitate one's participation in DSM activities and integrate them with their daily routines, was feasible and acceptable to be further evaluated on a large sample of people with diabetes^11^. The present study aimed to preliminary evaluate this intervention in people with diabetes type-2 aged 30–65 years old and achieved exciting results that are the focus of this article.

## Results

### Participants' characteristics and their follow-ups

Table 1 presents the characteristics of the participants. The mean age of participants in the intervention and control group was 57 ± 5.22 years and 55.2 ± 7.15 years, respectively. The percentages of the male subjects were 66.7% in the intervention and 46.7% in the control group. The mean hemoglobin A1C (HbA1C) level in the intervention group was 8.58 ± 0.99%, while it was 8.74 ± 0.88% in the control group. Preparing and eating healthy foods was also the main priority for the daily activity in both groups. Statistical tests, such as Chi-square, Fisher's exact, and independent *t*-test confirmed the homogeneity of the participants’ characteristics between both groups.

**Table 1:** Characteristics of the participants.

Variables	Intervention group (n = 15)	Control group (n = 15)	P value
Age (years)	57 ± 5.22	55.2 ± 7.15	0.438
Gender			
Female	5 (33.3)	8 (53.3)	0.462
Male	10 (66.7)	7 (46.7)	
Education			
High school or less	6 (40)	6 (40)	0.645^a^
Diploma	6 (40)	7 (46.7)	
Associate degree	1 (6.7)	1 (6.7)	
Bachelor's degree	1 (6.7)	0 (0)	
Master's degree	1 (6.7)	1 (6.7)	
Body Mass Index (kg/m^2^)	27.92 ± 2.76	28.68 ± 3.53	0.519
Diabetes duration (years)	10.26 ± 6.36	11.73 ± 5.89	0.518
Hemoglobin A1C (%)	8.58 ± 0.99	8.74 ± 0.88	0.646
Treatment regimen			
Oral medication	9 (60)	6 (40)	0.070
Insulin	6 (40)	9 (60)	
Priorities of daily activity performance			
1. Preparing and eating healthy foods	9 (60)	13 (86.7)	0.420
2. Doing exercise and physical activity	6 (40)	2 (13.3)	

The results are shown as means ± standard deviations or numbers (percentages).

P value: The results of the Chi-square test, Fisher's exact test, or independent t-test to examine the homogeneity of qualitative and quantitative variables between the groups.

^a^The Comparison between the number of people with a high school degree and less with the number of people with a diploma degree and more.

**Figure 1:**
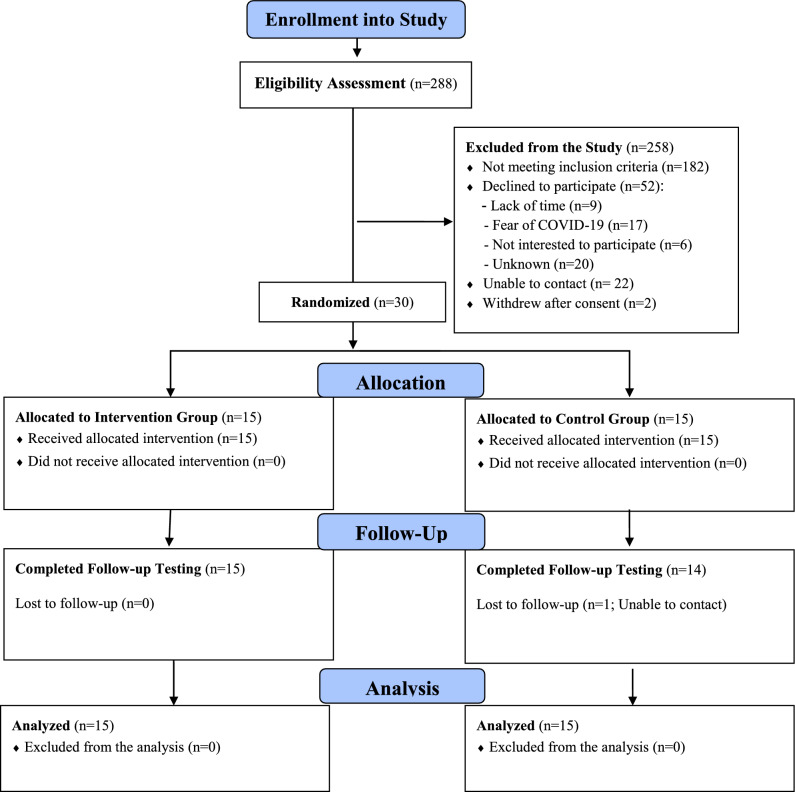
nnOverview of the participants and their follow-up process.

Figure 1 outlines an overview of participants’ follow-up. All of the participants received their assigned interventions, with 29 out of 30 (96.66%) completed their final assessments at the T3 time point. Among those participating in the OTDSM sessions, 13 (86.66%) completed all of the group meetings and individualized visits or phone calls. Also, 12 subjects (80%) completed all of the phone calls sessions in the control group. Tables 2 and 3 show the between-group comparisons among the study variables. Both Mann–Whitney U and Independent *t*-tests revealed no significant differences between groups for any of the variables before the intervention started.

**Table 2:** Between-group comparisons for biological parameters.

Variables	Group	T1		T3		Group effect	Time × group effect
		Mean ± SD	P	Mean ± SD	P value	P value (effect size)	P value (effect size)
Hemoglobin A1C	Intervention	8.58 ± 0.99	0.646	7.31 ± 0.89	**0.014**	**0.046 (**0.150)	0.180 (0.070)
	Control	8.74 ± 0.89		8.16 ± 0.77			
Fasting blood sugar	Intervention	161.87 ± 68.49	0.404	128.54 ± 34.31	0.125	0.766 (0.004)	**0.031 (**0.180)
	Control	144.73 ± 37.88		160.15 ± 62.89			
Triglyceride	Intervention	158.87 ± 71.17	0.315	142.54 ± 88.04	0.977	0.590 (0.010)	0.217 (0.060)
	Control	134.13 ± 60.85		143.38 ± 52.88			
Total cholesterol	Intervention	155.20 ± 48.64	0.586	159.00 ± 54.65	0.380	0.377 (0.030)	0.988 (< 0.001)
	Control	145.80 ± 44.77		144.38 ± 22.03			
High-density lipoprotein	Intervention	45.40 ± 7.82	0.547	45.08 (8.01)	0.695	0.560 (0.010)	0.794 (0.020)
	Control	47.47 ± 10.54		46.69 ± 12.30			
Low-density lipoprotein	Intervention	115/00 ± 38.72	0.320	99.31 ± 39.14	0.193	0.124 (0.010)	0.506 (0.003)
	Control	100.13 ± 41.64		88.40 ± 16.94			

Significant values are in [bold].

*T1* before the intervention, *T3* three months after the T1.

**Table 3:** Between-group comparisons for self-reported parameters.

Variables		Group	Independent T test or Mann–Whitney U test						ANOVA with repeated measures	
			T1		T2		T3		Group effect	Time × group effect
			Mean ± SD	P value	Mean ± SD	P value	Mean ± SD	P value	P VALUE (EFFECT SIZE)	P value (effect size)
DSMQ-total score		Intervention	5.98 ± 1.01	0.758	7.37 ± 1.11	**0.032**	7.33 ± 0.83	**0.012**	0.082 (0.126)	**0.01 (**0.181)
		Control	5.82 ± 1.62		6.11 ± 1.74		6.06 ± 1.62			
DSMQ-Subscales	Blood glucose management	Intervention	5.95 ± 1.56	0.155	7.56 ± 1.62	0.179	7.85 ± 1.55	**0.009**	0.21 (0.070)	**˂ 0.001 (**0.273)
		Control	6.75 ± 1.4		6.61 ± 1.93		6.14 ± 1.74			
	Diet control	Intervention	5.17 ± 1.53	0.451	7.02 ± 1.38^b^	0.268	7.11 ± 1.94	**0.050**	0.19 (0.070)	0.361 (0.040)
		Control	4.55 ± 2.63		6.09 ± 2.08		5.26 ± 2.85			
	Physical activity	Intervention	6.58 ± 2.07	0.463	8.09 ± 1.77	0.055	7.92 ± 2.30^b^	0.187	0.137 (0.090)	0.280 (0.050)
		Control	5.81 ± 3.30		5.98 ± 3.47		6.02 ± 3.89			
	Health care use	Intervention	6.87 ± 1.75	0.061	6.94 ± 0.71	0.087	6.64 ± 1.23^b^	0.788	0.059 (0.150)	0.762 (0.010)
		Control	5.55 ± 1.92^b^		5.93 ± 1.83^b^		6.42 ± 1.96			
DMSES-Total score		Intervention	4.09 ± 0.44	0.378	4.35 ± 0.35	0.143	4.38 ± 0.39	**0.011**	0.137 (0.090)	0.084 (0.100)
		Control	3.89 ± 0.76		4.08 ± 0.56		3.87 ± 0.60			
DMSES Subscales	General nutrition	Intervention	4.18 ± 0.61	0.067	4.54 ± 0.46^b^	**0.006**	4.47 ± 0.49^b^	**0.014**	**0.012 (**0.250)	0.334 (0.050)
		Control	3.59 ± 1.01		3.81 ± 0.65		3.70 ± 0.79			
	Specific nutrition	Intervention	3.36 ± 0.77	0.582	3.73 ± 0.84	0.499	3.93 ± 0.72	0.065	0.647 (0.010)	0.091 (0.090)
		Control	3.52 ± 0.79		3.49 ± 0.95		3.32 ± 0.98			
	Blood glucose control	Intervention	4.40 ± 0.66	0.202	4.45 ± 0.45^b^	0.585	4.57 ± 0.57^b^	0.467	0.282 (0.050)	0.769 (0.010)
		Control	4.11 ± 0.54		4.25 ± 0.72		4.28 ± 0.92			
	Physical activity control	Intervention	3.88 ± 0.95	0.680	4.28 ± 0.66	0.359	4.15 ± 0.89	0.060	0.284 (0.050)	0.150 (0.080)
		Control	3.71 ± 1.20		3.99 ± 0.91		3.42 ± 1.10			
	Medical management	Intervention	4.78 ± 0.32	0.648	4.89 ± 0.27^b^	0.549	5.04 ± 0.37	0.079	0.103 (0.110)	0.122 (0.100)
		Control	4.48 ± 1.05^b^		4.83 ± 0.29		4.51 ± 0.91^b^			
DDS-Total score		Intervention	2.08 ± 0.77	0.543	1.69 ± 0.52	0.545	1.51 ± 0.34	**0.045**	0.262 (0.054)	0.402 (0.039)
		Control	2.26 ± 0.79		1.86 ± 0.89		2.04 ± 0.90			
DDS Subscales	Emotional distress	Intervention	2.06 ± 0.98^b^	0.303	1.64 ± 0.65	0.313	1.52 ± 0.49	0.155	0.246 (0.060)	0.988 (0.001)
		Control	2.47 ± 1.26		2.01 ± 1.18		2.03 ± 1.24			
	Physician-related distress	Intervention	1.96 ± 1.29	0.946	1.66 ± 1.09	0.943	1.54 ± 0.73	0.992	0.943 (0.001)	0.852 (0.010)
		Control	1.94 ± 1.10^b^		1.63 ± 0.73		1.54 ± 0.70			
	Regimen-related distress	Intervention	2.43 ± 0.83	0.914	1.86 ± 0.58	0.970	1.73 ± 0.46	0.086	0.561 (0.010)	0.208 (0.070)
		Control	2.47 ± 1.03		1.85 ± 0.91		2.22 ± 0.96			
	Interpersonal distress	Intervention	1.83 ± 1.09	0.344	1.76 ± 1.10	0.703	1.44 ± 0.67	0.097	0.292 (0.050)	0.554 (0.020)
		Control	2.22 ± 1.07		1.95 ± 1.41		2.24 ± 1.64			
PHQ		Intervention	8.36 ± 5.23^b^	0.878	5.46 ± 4.46	0.284	4.66 ± 3.11	**0.046**	0.226 (0.063)	0.342 (0.046)
		Control	8.80 ± 5.24		7.28 ± 4.19		7.64 ± 4.48			
COPM-Performance		Intervention	3.53 ± 2.24	0.894	6.80 ± 1.45	0.654	7.23 ± 1.25	**0.016**	0.478 (0.020)	**0.045 (**0.130)
		Control	3.43 ± 1.20		6.43 ± 2.67		5.18 ± 2.64			
COPM-Satisfaction		Intervention	2.93 ± 2.29	0.582	6.74 ± 1.89	0.989	7.66 ± 1.55	**0.005**	0.565 (0.010)	**0.015 (**0.170)
		Control	3.36 ± 1.74		6.75 ± 2.76		5.32 ± 2.53			

Significant values are in [bold].

*T1* before the intervention, *T2* one month after the T1, *T3* three months after the T1, *COPM* Canadian Occupational Performance Measure, *DSMQ* Diabetes Self-Management Questionnaire, *DMSES* Diabetes Management Self-Efficacy Scale, *DDS* Diabetes Distress Scale, *PHQ* Patient Health Questionnaire. ^b^Variables with abnormal distribution based on the Shapiro–Wilk test.

### Biological parameters

The study results demonstrated a significant difference between groups for the HbA1C levels after the intervention. The repeated measure ANOVA analyses showed significant effects of the group on HbA1C (P = 0.046). There was also a significant interaction effect of the group by time on fasting blood sugar (FBS) changes (P = 0.031); (Table 2). The results of the Bonferroni post-hoc test for the intervention group showed significant differences in HbA1C levels (P = 0.006) over the 3-month intervention (data not shown).

### Self-reported parameters

The findings indicated an increasing trend in the scores for diabetes self-management questionnaire (DSMQ) in both groups at T2 and T3 time points. Regarding diabetes management self-efficacy scale (DMSES), Canadian occupational performance measure (COPM)-performance, and COPM-satisfaction scores, there was an increasing trend in T2 for both groups. However, at the T3 time-point, an increasing trend was observed in the intervention group while there was a decreasing trend in the control group (P ˂ 0.05). Also, there was a decreasing trend in the diabetes distress scale (DDS) and patient health questionnaire (PHQ) scores at the T2 point for both groups. However, in the T3 time-point, a decreasing trend in the intervention group, an increasing trend in the control group, and a significant difference existed between the two groups (P ˂ 0.05) with respect to these variables (Table 3).

The results from the repeated measure ANOVA revealed a significant effect of group on DMSES-general nutrition (P = 0.012). Also, there was a significant interaction between groups versus time for DSMQ-blood glucose management, COPM-performance, and COPM-satisfaction. The independent *t*-test revealed a significant difference between the groups only at T3 time-point for these variables (Table 3).

The Bonferroni post-hoc test for the intervention group resulted in a significant difference in the T1–T2 and T1–T3 for the DSMQ, DSMQ-blood glucose management, DSMQ-diet control, DDS-regimen, COPM-performance, and COPM-satisfaction. There was also a significant difference for the DSMQ-diet control in T1–T2 and DDS total score inT1–T3. In the control group, a significant difference in T1–T2 was explicit for COPM-performance, COPM-satisfaction, and DSMQ-diet control. There was also an increasing trend in the means for COPM-performance, COPM-satisfaction, and DDS-regimen in T1–T3. The results of the Bonferroni post-hoc test revealed no significant difference in DMSES and PHQ scores for both groups (data were not shown).

## Discussion

The present study showed that the OTDSM can significantly improve blood glucose levels in people with type-2 diabetes. The protocol achieves this by emphasizing people's participation in self-management activities and by integrating them into their daily routines. Previous studies have highlighted the effect of self-management on improving blood glucose in people with type-2 diabetes^12,13^ and reported an average decrease of 0.76% in HbA1C at immediate follow-up^13^. The present study found a 1.27% decrease in HbA1C immediately at the follow-up of people who were trained in the use of our self-management protocol (OTDSM). This achievement is comparable to the extent of glycemic target gained through the use of medications^12^. Implementing this protocol by people with diabetes type-2 is likely to result in reductions in diabetes complications and the various burdens the disease imposes on the individual and societ^14^.

Similar protocols introduced previously by Rovner et al. and Schepens Niemiec et al. improved blood glucose levels in elderly people and adults with type-2 diabetes^7,8^. Conversely, Pyatak et al. reported an increase in HbA1c levels of young people with type-2 diabetes who participated in an occupational therapy diabetes management program^6^. The contradiction may be due to the difference in the subjects being at young ages.

Based on our findings, the effects of our proposed protocol on self-management behaviors were observable. The self-management behaviors are important determinants of the disease control in diabetes. According to previous studies, the participation of people with diabetes in activities, such as blood glucose monitoring and recording, are the distinct differences between poorly managed (HbA1C ≥ 9.0%), moderately controlled (7.6 < HbA1C < 8.9%), and optimal (HbA1C ≤ 7.5%) blood glucose levels. Also, the extent to which an individual participates in dietary management activities distinguishes between poorly managed and moderately controlled diabetes. Furthermore, regular participation in exercise and physical activity distinguishes between moderately managed and optimal blood glucose levels^15^. The findings of this study demonstrated that the participants in both intervention and control groups had the lowest score on their dietary control subscales. Their main daily goal was focused on preparing and eating healthy foods. Results from the between-group comparisons of self-management behaviors at different time points showed that our proposed self-management protocol compared to offering DSM education only, improved self-management behaviors, blood glucose monitoring, and dietary control of people with type-2 diabetes as evident by the follow-up assessment data. This invaluable finding is likely to be associated with improvements in the subjects’ blood glucose levels, which was confirmed by the laboratory evaluations of their blood samples.

Our proposed self-management protocol also improved the participants' performance and satisfaction with their daily activities. In line with these results, earlier studies have also emphasized the effect of occupational therapy self-management interventions on daily activities in people with different chronic diseases^16–18^, including diabetes^8–10^. The most important feature of our self-management protocol was its client-centered approach and the focus on integrating self-management into people's daily lives, emphasizing increased diet control and physical activities based on their factual goals and priorities. These characteristics may affect one's perception of their daily activities^19^.

The present study demonstrated that our self-management protocol significantly improved self-efficacy in people with type-2 diabetes, especially with respect to consuming healthy foods.

Self-efficacy is a mental concept that is dependent on the situation and associated with the better achievement of desired outcomes in one’s life. Peer modeling, receiving social or individual support and encouragement from others, and emotional and physiological arousal are also significant factors in enhancing people's self-efficacy in performing desired tasks^20–22^. Our proposed self-management protocol focused on one's performance in their activities to achieve desired goals. It also had the advantages of both individual and group intervention structures. Combining these aspects, the possibility of one's family members to participate in the program, establishing communication and receiving social support among peers, dynamic and continuous communication of the intervention provider (IP) with the participants, and the use of cognitive, behavioral, and motivational techniques may influence one's self-efficacy in diabetes management. Emotional control and interpersonal relationships were also important areas of the proposed protocol that can improve the self-efficacy of the participants.

Previous studies have reported conflicting information about the effectiveness of occupational therapy interventions on self-management and self-efficacy of people with diabetes. In line with our findings, some studies have acknowledged that occupational therapy self-management interventions can impact self-efficacy positively in the management of other chronic diseases including type-2 diabetes^9,10,16,18^. Some of these interventions also improved exercise behaviors in people with type-2 diabetes^9,10^. In contrast, occupational therapy diabetes management intervention in some previous studies failed to improve the subjects’ self-management behaviors and self-efficacy^6,7^. These contradictory findings may be due to differences in the study designs, the interventions’ structures, the populations under study, and the tools used to measure the variables.

The current study showed that the OTDSM could not improve diabetes distress and depressive symptoms, as compared to general self-management education. However, the variables’ scores in those who received the protocol decreased over time, with a significant between-group differences at the T3 time-point. Consistent with our results, occupational therapy self-management interventions in previous studies also failed to lower diabetes distress in their study populations^6^ and the depressive symptoms in people with other chronic diseases^6,16,18^. Based on the available evidence, participating in self-management activities and promoting a healthy lifestyle can affect the psychological symptoms in people with diabetes^23,24^. The content of the OTDSM defined the desirable interventions for improving the distress and associated symptoms. However, the outbreak of Coronavirus disease (COVID-19) limited the number of intervention sessions that focused on controlling emotional and interpersonal relationships. The findings of the present and previous studies^6,16,18^ further showed mild depression in their participants which was present before the interventions began^25^. It is difficult to demonstrate the positive effects of the intervention if people have mild depression. Controlling negative emotions also is a time-consuming and complex process that requires more studies before an effective protocol is achieved^24^.

To date, little research has been conducted to incorporate DSM with people's daily routines. The present study is one of the pioneers in integrating DSM with the daily activities of adults with type-2 diabetes based on a robust, evidence-based intervention. The other strengths of this study include its methodological design, randomization, the high attendance rate of the participants, and the consideration of a wide range of outcomes. However, the study's small sample size causes some uncertainty in interpreting the findings. Future studies can conduct further investigations with a larger sample size. The other limitation of this study is the participants being from a single center which limits the generalizability of the results. Also, the study did not have the resources to plan a long-term follow-up to assess the lasting effect of the findings. Furthermore, the outbreak of COVID-19 limited the number of group sessions and the possibility of conducting face-to-face interventions.

## Methods

### Study design

The study was a single-center, randomized controlled trial conducted from July to December 2020. The ethics committee of the Iran University of Medical Sciences approved the study (IR.IUMS.REC1396.9321525002) and all procedures were performed by relevant guidelines and regulations. The research was also registered in the Iran Clinical Trials Center (IRCT20180105038224N1) on 02/12/2018.

### Participants

To recruit the participants, we used a simple non-probability sampling method by preparing a list of patients with diabetes who had been admitted to the Institute of Endocrinology and Metabolism affiliated with the Iran University of Medical Sciences, Tehran, Iran. Then, we invited the eligible individuals to participate in the study by phone calls. The inclusion criteria were: a diagnosis of type-2 diabetes for at least 1 year, being 30–65 years old, and absence of disabling comorbidities that prevented them from participating in the study. Also, the subjects needed the ability to read and write and showing a HbA1C level higher than 8% based on the laboratory test performed over the previous month. Any occurrence of acute medical conditions or the unwillingness of the participants to continue their participation in the study was the criteria for their exclusion from the research. Written informed consent was obtained from all participants before entering the study.

### Randomization and subject allocation

Eligible participants were randomly assigned equally to either an intervention or a control group (N = 15), based on a stratified permuted block randomization from a table of random numbers. Stratification was based on gender and age (≤ 47 years or ≥ 47 years). The research team concealed the intervention plan from the person who assessed the study outcomes.

### Intervention

#### The OTDSM intervention

##### Interventionstructure and schedule

The intervention group received the OTDSM which involved a combination of individual and group sessions, both face-to-face, and remote ones. The program started with an individual face-to-face session lasted about 45 min, then continued with four face-to-face group meetings took approximately 120–150 min, and five individualized and mostly remotely ones provided weekly over ten consecutive weeks. The face-to-face sessions were held at the School of Rehabilitation of Iran University of Medical Sciences, while the remote sessions were conducted via phone calls. The participants' family members could also participate in the program, if necessary.

The outbreak of COVID-19 at the time of the study caused the research team to provide the participants transportation to attend the in-person sessions. The subjects in the intervention group were also randomly divided into two subgroups of 7 and 8 persons each. These subgroups had their meetings separately in a large hall.

##### Intervention contents

A Ph.D. candidate in occupational therapy with ten years of clinical experience provided the intervention. The program started out with an individual face-to-face session, during which the IP used a collaborative and client-centered approach to establish a proper therapeutic relationship with the participants. The main focus was to identify the subjects’ concerns and priorities and review their daily activities. This session continued with counseling and motivational interviewing to trigger the individuals’ motivation to improve upon their daily routines. The evidence-based educational contents of the intervention^26,27^ were then shared with the subjects during four consecutive face-to-face group sessions. These included preparing and eating healthy foods, exercising and physical activity, taking medication, caring for their feet, doing regular follow-ups, monitoring self for blood glucose levels and diabetes complications, controlling one’s emotions, expanding social networks, and having or developing intimate relationships. The teaching materials included a PowerPoint presentation with clear illustrations and highlighted texts plus instructional handouts. The group meetings also let the IP facilitate the participants' sharing of their DSM participation experiences with peers, improving their motivation to plan new daily routines and learn how to solve their problems.

Based on the provided information on each session, the participants set their goals toward their participation in DSM activities. The subjects then wrote an activity diary to include their individual goals and implement the new daily schedules. Accordingly, they practiced the planned health-related activities, adjusted their daily routines, and tried to develop new habits to make progress and succeed in DSM activities. To facilitate the subjects’ participation, the IP continuously reviewed the individuals’ activity patterns during the group and individual sessions. Accordingly, she applied appropriate strategies to facilitate their participation in DSM^11^. Table 4 describes the intervention contents and strategies and provides examples of the participants' practiced skills.

**Table 4:** The content of the OTDSM intervention.

Content areas	Contents	Strategies	Examples
Doing regular follow-ups, self-monitoring, and daily care	Knowledge about diabetes, its' symptoms, complications, and routine care The important role of doing regular follow-ups, self-monitoring, and daily care in the control of diabetes and related complications Setting goals related to the care of feet, regular use of medications, SMBG, and SMDC Reschedule daily routines regarding one's occupational participation in the care of feet, use of medications, SMBG, and SMDC, creating new habits, and integrating these with their daily lives	Therapeutic use of self Establishment of therapeutic relationships with the participants Identifying the participants' patterns of daily activities Identifying the participants' self-management priorities and problems	After writing about daily activities in an activity diary, Mr. H realized that one of his diet mistakes, which caused him to overeat at meals, was to skip snacks. With the education and counseling provided by the IP about the importance of regular meals in diabetes management, Mr. H incorporated morning and evening snacks into his activity diary. By practicing and participating in this activity, attending group meetings, problem-solving with peers, and receiving phone call reminders from the IP, Mr. H practiced integrating this activity into his daily routines
Eating healthy foods	Awareness of the do's and do nots of nutrition, eating time, and the importance of regular meals Setting goals related to eating healthy foods Reschedule daily routines regarding one's occupational participation in the preparation and eating of healthy foods, how to create new habits, and integrate these with their daily lives	Motivational interviewing Individual counseling Using educational tools	
Doing regular exercise and physical activity	Awareness of the importance of exercise in the control of diabetes, appropriate exercise programs, and exercise precautions in people with diabetes Setting goals related to doing regular exercise and physical activity Reschedule daily routines regarding one's participation in doing regular exercise and physical activity, how to create new habits, and integrate these with their daily lives	Using goal-setting forms Using activity diaries Discussion and problem-solving in the groups	Mrs. A concerned about her little exercise due to the closure of gyms after the outbreak of COVID-19. In group and individual intervention sessions, she learned to think of new ways to solve exercise obstacles, choose the best method that fits her situation, and apply it. She planned to walk with her husband one day a week and exercise at home two days a week. Accordingly, she introduced new activities in her activity diary and developed a new exercise routine through practice and repetition. Her wife's support, sharing this experience with peers, and continuous interaction with the IP facilitated Mrs. A to continue her new daily plans
Control of emotions and interpersonal relationships	Awareness of the importance of the control of emotions, intimate relationships, and interpersonal relationships in the management of diabetes Teaching coping strategies for the control of emotions and interpersonal relationships Setting goals related to the control of emotions and interpersonal relationships and practice related skills on a daily basis	Sharing self-management participation experiences in groups Phone calls and reminders Involvement of family members	

*OTDSM* Occupational Therapy Diabetes Self-Management, *SMBG* Self-Monitoring Blood Glucose, *SMDC* Self-Monitoring Disease Complications.

The group sessions were conducted followed by five individualized ones, mostly by phone calls. These sessions were conducted by the IP based on the final goal-setting plans and the daily schedules of each participant, which were given to the IP at the last group meeting. Each phone call lasted about 15 min, during which the IP reviewed the daily plan of each participant and answered their questions while noted the changes made in their plan. Accordingly, she guided the participants to establish new daily routines by repeating DSM activities. Two individualized face-to-face sessions were held as remote phone calls could not meet some of the needs of two of the participants; each lasted about 30 min.

##### Diabetes self-management education

The control group received an individually delivered educational program, provided at the Institute of Endocrinology and Metabolism of Iran University of Medical Sciences, Tehran, Iran. In this program, the IP handed an educational booklet to each participant and briefly reviewed its contents in 20 min. The booklet covered principles of nutrition, exercise, emotional management, diet, diabetes medication, and the need for regular follow-ups. Next, the IP called each of the participants once a week for three consecutive weeks to answer their questions and encourage them to engage in diabetes management activities.

### Outcomes

#### Biological parameters

All biological parameters were measured before the first intervention (T1) and three months later (T3) by collecting a 12-h fasting blood sample from each participant at the laboratory of the Institute of Endocrinology and Metabolism of Iran University of Medical Sciences. The primary data collected were the participants’ levels of HbA1C, measured on an ion-exchange chromatography equipment (DS5 Analyzer, Drew Scientific Limited, Cumbria, UK). Also, the FBS was measured on a glucose analyzer (YSI 2700 Select, YSI, Inc., Yellow Springs, OH). The subjects’ lipid profile was also measured on an auto-analyzer (Liasys, AMS, Italy). This included the blood levels of triglyceride (TG), total cholesterol (TC), high-density lipoprotein (HDL), and low-density lipoprotein (LDL).

#### Self-reported measures

The self-reported parameters were taken three times, once before the first intervention visit (T1), one month into the study (T2), which was at the end of face-to-face group sessions, and two months later (T3), which was at the end of the 10-week treatment period. These measures are as the following:

##### Self-management behaviors

A DSMQ^15,28^ was used to assess the subjects’ perception of their participation in activities related to diabetes management. The questionnaire covered four areas, such as blood glucose management, diet control, physical activity, and healthcare issues. It contained 16 items, with seven of them worded positively with the other nine worded negatively. The subjects rated each item based on a four-point Likert scale, from zero (not applicable to them) to three (very much applicable to them). The questionnaires were scored based on the following formula:

$$\mathrm{Self}-\mathrm{Management Behavior}=\frac{\mathrm{The sum of item score }}{\mathrm{Maximum possible score for the item}}\times 10$$

Accordingly, a score closer to ten was interpreted as being a more desirable self-management behavior than a smaller score^15^.

##### Self-efficacy in diabetes

The DMSES^29,30^, a 20-item questionnaire based on Bandura's self-efficacy concept, was used to assess the subjects’ confidence in their ability to manage diabetes. The participants rated each question based on a five-point Likert scale from one (no, surely not) to 5 (yes, surely) (29). The questionnaire’s scoring involved calculating the mean total and the average scores for each subscale of specific nutrition, general nutrition, blood glucose control, physical activity and weight control, and medical management^30^.

##### Diabetes distress

The DDS^31,32^ is a 17-item questionnaire to assess the subject’s burdens and worries of living with diabetes and its' management. The questionnaire contained four subscales to measure negative emotional burdens (emotional subscale), concerns about having a trusted physician and receiving appropriate support and care (physician subscale), worries related to diet, physical activity, and medication (diet subscale), and concerns about receiving appropriate support from family and friends (interpersonal subscale). The subjects rated each question on a six-point Likert scale, from one (not a problem) to 6 (serious problem)^33^. The scoring was done by calculating the mean total and the subscale scores. A score less than 2 indicated little or no distress, between 2 and 2.9 signified moderate distress, and 3 or higher indicated high distress^31^.

##### Depressive symptoms

The PHQ-8^34,35^ is an appropriate tool for identifying symptoms of depression in various populations, including people with diabetes. The subjects scored each of the eight questions on the following scale: 0 (not at all), 1 (several days), 2 (more than half the days), and 3 (nearly every day). The scoring system of this questionnaire is the sum of the scores for the questions, from zero to 24. A score between zero and four indicates no symptoms of depression, a score between 5 and 9 implies mild depression, a score between 10 and 14 signifies moderate depression, a score between 15 and 19 represents moderately severe depression, and a score between 20 and 24 indicates severe depression^25^.

##### Performance and satisfaction with daily activities

The COPM^19,36,37^ was used to assess the subjects’ perception of their performance and satisfaction with important daily activities, such as conducting one’s occupation. Using a client-centered perspective and through semi-structured interviews, this tool allows for a detailed assessment of daily activities in the areas of self-care, productivity, and leisure. The evaluation begins with an open-ended question asked of the subjects to explain what they do on a typical day and if there are any activities that they need or want to do and have difficulty doing. They then prioritize the most important problems in performing their daily activities and rate both their performance and satisfaction with every activity on a scale from one to ten. The scoring involves calculating the mean scores of performance and satisfaction with the activities determined by the individual. Accordingly, a higher score indicates better performance in daily activities and greater satisfaction with participation in these activities^19^. The scale has acceptable reliability among people with chronic diseases^37^ and noticeable content validity for its' Persian translation^36^.

### Statistical analyses

#### Power analysis

Thirty people with diabetes participated in this study, with 15 persons in each of the two groups. A one-tailed power calculation of the data collected at the T3 time point showed that the sample size was sufficient to maintain an 82% power and 95% confidence interval for detecting significant between-group differences for the primary outcome (HbA1C) at follow-ups compared to the baseline (G-Power software^38^).

#### Data analysis

To describe the quantitative and qualitative variables, respectively, the means plus standard deviations or the numbers plus the percentages were used. The normal distribution of the quantitative variables was examined by the Shapiro–Wilk test at a significance level of 0.01. The Chi-square test and Fisher's exact test were used to evaluate the homogeneity of the qualitative variables between the two groups. Independent *t*-tests and Mann Whitney U tests were used for between-group comparisons of the data collected at different time points. The repeated measures analysis of variance (ANOVA) was used for the between-group comparisons by matching the main effect of time with the interaction effect of the group by time. The Bonferroni post hoc test was used to obtain within-group pairwise comparisons at different time points. The data analysis was performed using the SPSS software, version 18, at a significance level of 0.05.

## Data Availability

The datasets used and analyzed during the current study are available from the corresponding author on reasonable request.
